# Preoperative Serum Triglyceride to High-Density Lipoprotein Cholesterol Ratio Can Predict Prognosis in Non-Small Cell Lung Cancer: A Multicenter Retrospective Cohort Study

**DOI:** 10.3390/curroncol29090481

**Published:** 2022-08-25

**Authors:** Junhong Li, Cong Ma, Xuhui Yuan, Xiaoyan Wang, Na Li, Ronghui Yu, Hui Liao

**Affiliations:** 1 Department of Surgery, Tongji Hospital, Tongji Medical College, Huazhong University of Science and Technology, Wuhan 430030, China; 2 Department of Surgery, First Affiliated Hospital of Nanchang University, Nanchang 330006, China; 3 Department of Surgery, Third Affiliated Hospital of Nanchang University, Nanchang 330008, China

**Keywords:** NSCLC, triglyceride, high-density lipoprotein cholesterol, prognosis

## Abstract

Background: Previously, research has reported associations of lipid and lipoprotein imbalances with carcinogenesis and cancer progression, so they have been considered as promising prognostic biomarkers for cancer in recent years. However, the correlation of preoperative serum triglyceride to high-density lipoprotein cholesterol ratio (TG/HDL-C) with non-small cell lung carcinoma (NSCLC) prognosis remains under exploration. Here, the study investigated the prognostic function of TG/HDL-C for NSCLC. Methods: The total combined group of this retrospective study enrolled 479 NSCLC patients from two tertiary referral hospitals, of which 223 patients were defined as the training group (Nanchang) and the remaining 256 were defined as the validation group (Wuhan). The cut-off of preoperative TG/HDL-C was determined through ROC curve in the training group and verified in the validation and combined groups subsequently. With one Cox proportional hazards model and K-M survival curves, a survival analysis was conducted. Results: In the training group, the optimal cut-off of TG/HDL-C was 1.02. Furthermore, the data based on the training group revealed a greater, shorter, overall survival (OS) in patients having a high TG/HDL-C (>1.02) than those having low TG/HDL-C (≤1.02). Meanwhile, in univariate and multivariate analysis, for prognostic OS among NSCLC patients, TG/HDL-C acted as one independent factor. All the results above were confirmed in the validation and combined groups. Conclusion: NSCLC patients with a comparatively low preoperative serum TG/HDL-C level had a correlation with well OS. TG/HDL-C possibly acted as one novel, effective prognostic biomarker for NSCLC patients.

## 1. Introduction

Lung cancer, the primary reason for global cancer-associated deaths, occupies over 120,000 deaths each year in the United States of America [1]. In China, lung cancer is the most frequently-seen cancer, with the highest mortality rate among all cancers [2]. Non-small cell lung cancer (NSCLC), primarily composed of lung squamous cell carcinoma, as well as adenocarcinoma, occupies about 85% of all lung cancer patients [3]. Surgical resection is the most effective therapy for stage I-II NSCLC and in a subset of cases with stage IIIA NSCLC [4]. By contrast, patients with stage IIIB–IV NSCLC are not amenable to curative resection; therefore, thoracic radiotherapy, chemotherapy, molecular targeted therapy, and immunotherapy are alternative treatment modalities for these patients [4]. Despite the availability of various treatment options, the overall survival (OS) rates of late-stage NSCLC cases in 5 years continue to remain poor (36% 5-year OS rate in stage IIIA compared to 83% in stage IA) [5,6]. The unsatisfactory prognosis is generally attributed to the difficulty in early diagnosis and the high propensity for metastasis [7]. Moreover, due to the heterogeneity of NSCLC, the traditional tumor-node-metastasis (TNM) staging system may not be an accurate tool for prognostic assessment of these patients, as patients at the same stage usually have different survival outcomes [8,9]. Owing to the poor long-term outcomes and the paucity of prognostic indicators, identification of more effective prognostic biomarkers for NSCLC is a key imperative to guide treatment decision-making.

Several recent studies have indicated potential roles of imbalance in serum lipid indices, such as high-density lipoprotein cholesterol (HDL-C), triglyceride (TG), as well as cholesterol, in the causation and prognosis of cardiovascular diseases and cancer [10,11,12]. TG, one of the components of serum lipids with pro-cellular proliferative activity, was shown to be linked with cancer cell proliferation and differentiation, and cancer-associated cell growth [13,14]. Cholesterol, an essential structural component of lipid rafts, maintains a vital part in maintaining the structural integrity of cell membranes as well as intracellular signal transduction [15]. HDL-C can remove cholesterol from peripheral tissues and reverse-transport it to the liver, and thus can be regarded as a potent antioxidant and anti-inflammatory factor [16]. The metabolism of serum lipids and lipoproteins, such as TG and HDL-C, has a strong correlation to the nutritional status of patients (BMI), diet, and stressful metabolic states [17]. Reportedly, abnormalities in serum lipids and lipoproteins are strongly bound up with the development as well as the progression of cancer, suggesting that their potential roles in carcinogenesis and providing an innovative theoretical basis for anticancer treatment [18,19,20,21]. Until now, some studies have identified reduced serum HDL-C levels as one risk factor for ovarian cancer, hepatocellular, and nasopharyngeal carcinoma [17,22,23]. Conversely, Wang et al. [24] found upregulated serum HDL-C as one risk factor for colorectal cancer. Thus, there is no clear consensus on the pathological relationship between dyslipidemia and cancer. Additionally, the correlation of TG and HDL-C levels with cancer prognosis is not well characterized, especially in the context of NSCLC. In only limited studies, serum TG or HDL-C was linked to the prognosis of NSCLC patients [25,26,27].

Interestingly, Sun et al. [28] found a higher sensitivity and predictive function of TG/HDL-C in the prognostic assessment for gastric cancer when compared with any single lipid parameter. They observed that, when compared with those of other serum lipids (TC, LDL-C, HDL-C, as well as TG), TG/HDL-C showed the largest AUC in independent forecasting of 5-year OS among gastric cancer patients [28]. Retrospective cohort research by Dai D. et al. also indicated TG/HDL-C as one independent predictive biomarker for OS among triple-negative breast cancer patients, with an advantage over the serum TG level [29]. Indeed, each single lipid parameter reflected only one aspect of interaction with cancer. Use of an index that combines multiple parameters may unravel the association between cancer and lipid metabolism with a higher accuracy and sensitivity [18,28,29]. According to previous research by Ma et al. [25], preoperative serum TG and HDL-C can be factors for independent outcome prediction among NSCLC patients. However, the link of TG/HDL-C with NSCLC prognosis remains unknown, which is also the original intention of our current research. 

Therefore, with a multicenter retrospective cohort design, this study was for investigating the prognostic function of preoperative TG/HDL-C among NSCLC patients.

## 2. Methods

### 2.1. Study Population

The Ethics Committee of Tongji Hospital of Tongji Medical College of Huazhong University of Science and Technology, as well as First Affiliated Hospital of Nanchang University approved this retrospective research (reference number: TJ-IRB20220802). A total of 479 NSCLC patients from these two tertiary referral hospitals were enrolled and further defined as the training group (Nanchang, *n* = 223) or the validation group (Wuhan, *n* = 256), respectively. The inclusion criteria: cases ≥18 years who were pathologically diagnosed as NSCLC (stages I-IIIA); availability of complete clinical data; blood samples were collected before therapy. The exclusion criteria: (1) patients who had received neoadjuvant chemoradiotherapy; (2) those with malignancies that originated from any organ other than the lung; (3) those receiving any drug that affected lipid metabolism; (4) presence of concomitant diseases associated with deranged lipid metabolism, such as hyperlipidemia, diabetes, or metabolic syndrome; (5) history of blood transfusion in the four-month period immediately preceding the hospital admission.

Patients in the training group were diagnosed and treated in the First Affiliated Hospital of Nanchang University between March 2013 and September 2018. Patients in the validation group were diagnosed and treated at the Tongji Hospital of Tongji Medical College of Huazhong University of Science and Technology from October 2014 to October 2019. The diagnosis and stage of NSCLC were determined in accordance with the criteria of the Union for International Cancer Control (2017 UICC-8 criteria). According to the perioperative management principles, every patient was asked to stop smoking and alcohol consumption within 14 days before operation, and were given a light diet. The therapy protocols of 479 patients were developed and implemented under the instructions from the National Comprehensive Cancer Network.

### 2.2. Follow-up

Every patient was followed up under the recommendations of the 2017 UICC-8. Follow-up was made to patients who were discharged after therapy every 3 months for the first 2 years, and every 6 months for the next 3–5 years, and then annually thereafter. In the training group, follow-up ended on patient death or 30 November 2020, and in the validation group, the follow-up terminated on 30 November 2021, or death.

### 2.3. Statistical Processing

Statistical processing was performed via IBM SPSS 23.0 (SPSS, Chicago, IL, USA) as well as GraphPad Prism 7.00 (GraphPad Software, La Jolla, CA, USA). The Kolmogorov-Smirnov test was adopted for assessing the distribution normality of continuous variables. Those in normal distribution were reported by mean ± SD, and others were presented as median (first-third interquartile range [IQR]). Categorical variables (frequency (%)) were assessed using the chi-squared test. Receiver operative characteristics (ROC) curve analysis was performed for determining optimal cut-off and the area under the ROC curve (AUC) of preoperative TG/HDL-C in OS prediction. Survival analysis was conducted using K-M curves and the independent prognostic factors were determined though a Cox proportional hazards model. Two-sided *p* values < 0.05 suggested a notable difference.

## 3. Results

### 3.1. Patients’ Characteristics in Two Independent NSCLC Cohorts

Table 1 depicts patients’ detailed baseline characteristics. A total number of 479 patients with NSCLC which met our inclusion criteria were identified and enrolled here, including 223 and 256 in the training group and validation group, respectively. For the total combined group, 150 (31.3%) were women and 329 (68.9%) were men. The number of NSCLC patients accepted adjuvant chemotherapy in the training and validation groups, respectively, were 116 (52.0%) and 161 (62.9%). In the training group, 125 out of 223 patients survived at the last follow-up, 104 deaths were found during the whole follow-up in the validation group. Therefore, the OS ratios were 56.1% and 59.4% for training and validation groups, respectively. The baseline clinicopathological parameters in both cohorts were comparable.

### 3.2. Identifications of Optimal Cut-Off Value of TG/HDL-C Derived from the Training Group

We identified the optimal cut-off of TG/HDL-C and the corresponding AUC in NSCLC via ROC curve-based analysis. Using OS as the primary treatment outcome in the training group, the optimal cut-off of TG/HDL-C having the highest sensitivity and specificity was 1.02 (*p* = 0.005, AUC: 0.632, 95% CI 0.559–0.706). In the light of the optimal cut-off, the patients were assigned to high TG/HDL-C (TG/HDL-C >1.02) and low TG/HDL-C group (that ≤ 1.02).

### 3.3. Relation of the TG/HDL-C with Multiple Clinicopathologic Values

The relations of the TG/HDL-C with various clinicopathologic parameters were shown in Table 2. High or low group of TG/HDL-C levels were greatly different with pN in the training group (*p* = 0.002), validation group (*p* = 0.006), as well as combined group (*p* < 0.001). Moreover, different TG/HDL-C groups had strong relations with histological type in the training cohort (*p* = 0.038). 

### 3.4. Prognostic Value of Preoperative TG/HDL-C in the Training Cohort

**Figure 1:**
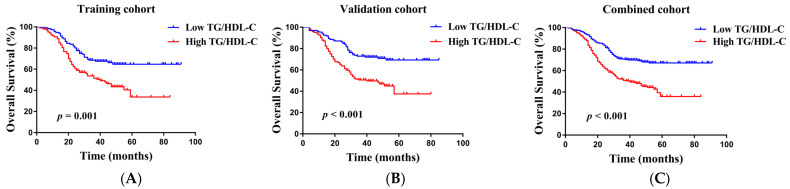
The Kaplan–Meier curves analysis for OS in NSCLC patients stratified by TG/HDL-C from the training (**A**), validation (**B**), and combined cohorts (**C**).

For further determination of the significance of TG/HDL-C for prognostic NSCLC, we next plotted K–M curves and performed univariate and multivariate Cox proportional hazards models using data from the training group. Patients having high TG/HDL-C presented much shorter OS than those having low TG/HDL-C in the training group (Figure 1A). Univariate Cox proportional hazards model revealed a significant association of stage, pT, pN, adjuvant chemotherapy, surgery type, as well as TG/HDL-C with OS in the training group (all *p* < 0.05, Table 3). Multivariate Cox proportional hazards model identified TG/HDL-C as one factor for independent OS prediction in the training group (HR: 1.674, 95% CI 1.094–2.559, *p* = 0.017).

### 3.5. Verification of the TG/HDL-C in the Validation Group and Combined Group 

K–M analysis revealed a relation of low TG/HDL-C with longer OS in the validation group (Figure 1B). In Table 3, the univariate model revealed a significant association of TG/HDL-C with OS in the validation group (HR: 2.236, 95% CI 1.499–3.336, *p* < 0.001). Moreover, the multivariate model further identified TG/HDL-C as one factor for independent OS prediction in the validation group (HR: 1.770, 95% CI 1.173–2.672, *p* = 0.007).

We combined the training and validation groups into a joined group for further survival analysis (Table 4). As indicated in Figure 1C, patients having high TG/HDL-C had a poor OS than those having low TG/HDL-C. According to univariate analyses, pT, stage, pN, surgery type, adjuvant chemotherapy, and TG/HDL-C were bound up with OS (all *p* < 0.05). According to multivariate analyses, pN (*p* < 0.001), stage (*p* < 0.001), adjuvant chemotherapy (*p* < 0.001), surgery type (*p* = 0.046), and TG/HDL-C (*p* < 0.001) were factors for independent OS prediction of NSCLC patients. Thus, the results of validation and combined groups were similar to those obtained from the training group.

### 3.6. Predictive Performance of TG/HDL-C for NSCLC

**Figure 2:**
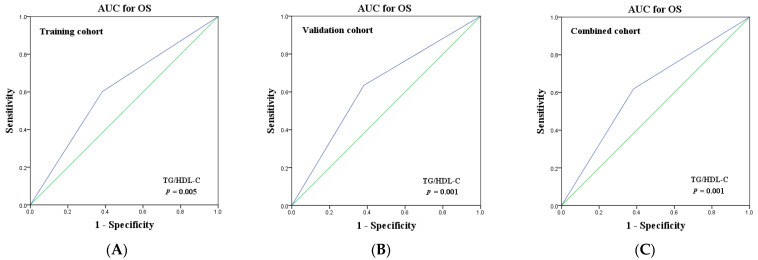
Prognostic predictive ability of TG/HDL-C for NSCLC patients by ROC curves in the training (**A**), validation (**B**), and combined (**C**) cohorts.

We further investigated the prognostic function of TG/HDL-C using the AUC calculated by ROC curve. According to Figure 2, the AUC for TG/HDL-C was 0.632 (95% CI 0.559–0.706, *p* = 0.005), 0.656 (95% CI 0.589–0.724, *p* = 0.001), as well as 0.644 (95% CI 0.594–0.693, *p* = 0.001) in the training, validation, as well as combined groups, respectively. Collectively, these findings revealed that, with respect to predicting OS for patients with NSCLC for prognosis, TG/HDL-C has a good performance.

## 4. Discussion

This study assessed the prognostic function of TG/HDL-C in the training, validation, as well as combined groups of NSCLC patients with stages I-IIIA. We found that relatively low preoperative serum TG/HDL-C (≤1.02) in NSCLC patients was associated with favorable OS. More importantly, preoperative serum TG/HDL-C was one independent factor for OS predication in NSCLC case according to findings.

These current findings were in accordance with several previous studies. To date, limited studies indicated TG/HDL-C as one independent predictor and its inverse association with the prognosis of gastric and triple negative breast cancer [28,29]. However, the cut-off value in this multicenter retrospective study (1.02) was different from that in triple negative breast cancer (0.600) and gastric cancer (1.20), which may be attributed to differences in cancer type, cancer stage, and number of patients. A retrospective study of Ma et al. [25] proved the association of the preoperative upregulated TG and downregulated HDL-C with an unfavorable prognosis in patients who died from NSCLC. Furthermore, Luo et al. [26] reported that relatively high HDL-C before adjuvant chemotherapy were independent prognostic factors with longer disease-free survival for NSCLC patients. Meanwhile, Lv et al. [30] revealed that the decrease in HDL-C level could indicate liver dysfunction caused by adjuvant chemotherapy in NSCLC, which partly supported the predictive significance of TG/HDL-C for patient prognosis. Additionally, prior research had also shown that lipid imbalances, including elevated TG levels or decreased HDL-C levels, enabled to predict a poorer prognosis in ovarian, colorectal, and breast cancer [17,31,32].

De novo lipogenesis and exogenous lipid uptake were the two important sources of lipids for cancer cells, and lipid levels in cancer were determined by both of these factors [33]. McLaughlin et al. [34] discovered the ability of TG/HDL-C to serve as one alternative biomarker of insulin resistance, which further stimulated TG production through lipolysis and de novo lipogenesis in the liver.

Our findings showed the ability of preoperative TG/HDL-C to act as one independent biomarker in OS prediction of patients with NSCLC. The prognostic function of TG/HDL-C in NSCLC cases was possibly attributable to the following reasons. For nearly two decades, a mountain of evidence has implicated both oxidative stress and chronic inflammation as pivotal factors regarding the causation of carcinogenesis and cancer progression [35,36,37]. Sustained oxidative stress induced chronic inflammation by activating the inflammation-associated transcription factors such as NF-κB, p53, PPAR-γ, AP-1, and Nrf2 [35]. The subsequent chronic inflammation induced transformation of normal cells into cancer cells through the activation of inflammatory signaling pathways [36]. An increasing body of evidence suggested a pro-carcinogenic effect of upregulated TG and downregulated HDL-C, due to their close involvement in oxidative stress and chronic inflammation [25]. Reactive oxygen species (ROS)-induced oxidative stress can upregulate TG levels, and high TG levels further induce carcinogenesis, for example, by activating the MAPK and PI3K/AKT signaling pathways [38]. Furthermore, TG-rich remnant also stimulated the growth and proliferation of cancer cells through G protein-coupled receptor [39]. Of note, in the study by Koohestani et al. [40], high serum TG level was found to promote colon tumorigenesis in high-fat diet rats as compared to those having normal TG levels. In contrast, Wang et al. [41] found that nude mice having low serum TG levels induced by low-fat diet presented strong inhibition of the growth of human prostate LNCaP tumor cells. Furthermore, clinical research had demonstrated the correlation of high TG level with increased morbidity and poor prognosis in NSCLC patients, also suggesting the pro-cancer potential of TG [25,42]. 

Cholesterol metabolites had also been implicated in the development of various cancers [15]. Mitochondrial cytochrome P450 family enzymes metabolize cholesterol to synthesize steroids, such as estrogen [15,43]. Estrogen can stimulate cancer cell proliferation in NSCLC patients through genomic and non-genomic signaling pathways [44]. Moreover, Wang et al. [45] found that the accumulation of intracellular cholesterol in KRAS^G12C^ inhibitor-treated NSCLC cells may result in adaptive resistance of tumors against targeted MAPK pathway inhibitors. Conversely, HDL-C removed cholesterol from peripheral tissues by reverse transporting cholesterol to the liver, and thus could be regarded as a potent anti-cancer factor [16]. HDL-C could exhibit anti-inflammatory, anti-proliferative, and anti-oxidant properties, thereby exerting anticancer potential in the tumor microenvironment [46,47]. HDL-C acted as an antioxidant by hydrolyzing oxidized lipids and preventing lipid peroxidation in biological membranes via the enzyme paraoxonase-1 [48]. Additionally, HDL-C was able to suppress the elevation of pro-inflammatory factors and adhesion molecules such as tumor necrosis factor α (TNF-α), interleukin-1β (IL-1β), IL-6, as well as intercellular adhesion molecule-1, thereby enhancing its anti-inflammatory effect [47]. Furthermore, multiple overlapping mechanisms were found to mediate the anti-inflammatory effect of HDL-C in several cell types [49]. Therefore, reduced HDL-C level in the setting of oxidative stress and chronic inflammation also impaired its above-mentioned functions, thereby establishing a pro-cancer microenvironment. Overall, higher TG level and lower HDL-C level may potentially indicate the progression of inflammation and oxidative stress in the tumor microenvironment, and thus help predict poorer clinical outcomes in cancer patients [17,22].

## 5. Strengths and Limitations

It is noteworthy that the present study first unveiled the ability of preoperative serum TG/HDL-C level to serve as an independent prognosis factor in NSCLC. Clinically, TG/HDL-C was probably considered as a valuable predictive biomarker that could determine NSCLC patients with shorter OS, thus enhancing the quality of life for the special patient groups through modifying individualized clinical therapy and strengthening the surveillance.

The strength of the present study was that the hematological parameters involved in the TG/HDL-C used to predict NSCLC prognosis were easily obtained from preoperative routine serum tests. Compared with traditional TNM staging system, serum lipids and lipoproteins had the characteristics of routine preoperative collection, cheap and non-invasion, which made TG/HDL-C more suitable for early preoperative evaluation of prognosis in developing countries. Additionally, TG/HDL-C combining information from two negatively correlated variables may have been more meaningful as a biomarker, with superior attributes of simplicity compared with any single lipid indicator [25]. TG/HDL-C reflected the TG and HDL-C levels and contained the interrelationship between the two factors, thus the ratio had a higher sensitivity and predictive significance for assessing the prognosis in cancer when compared with any single lipid parameter, such as TG, TC, HDL-C, as well as LDL-C [28]. Moreover, our study was designed as one multi-center study, and the patients enrolled in the training and validation group were from two tertiary referral hospitals in Wuhan and Nanchang, respectively. Compared with the more common single-center retrospective studies in the past, the current study tried to avoid the potential bias. Further, the present multi-center study included a larger sample size than other similar studies.

This research still had some shortcomings. First, in the retrospective research with a comparatively limited sample size, the impact from selection bias cannot have been ruled out. Secondly, although we demonstrated the prognostic value of TG/HDL-C, we did not compare its predictive ability with that of other known prognostic markers. Third, owing to the small number of cases of large cell lung cancer included in this study, our findings may not have been entirely representative of the clinical status of patients with this histologic type. Lastly, due to differences in patient numbers and cancer stage, the cut-off value of TG/HDL-C varied in prior research. Large-scale prospective studies are needed for determining the unified TG/HDL-C cut-off value and verifying our results. Furthermore, basic experiments are needed for unraveling the pathological mechanism among TG, HDL-C, and NSCLC.

## 6. Conclusions

In conclusion, our findings first evaluated and demonstrated the prognostic function of preoperative serum TG/HDL-C levels for NSCLC. Low TG/HDL-C level likely acts as a novel and effective prognostic biomarker of favorable OS in NSCLC patients. Clinically, determining lipid biomarkers associated with NSCLC prognosis can be of great importance to predict the survival, guide individualized clinical therapy, and to improve outcomes.

## Figures and Tables

**Table 1:** The clinical characteristics of subjects in the training, validation, and combined cohorts.

Characteristics	Training Cohort		Validation Cohort		Combined Cohort	
	*N* = 223	%	*N* = 256	*%*	*N* = 479	%
Age (years)	60.80 ± 10.75		59.91 ± 10.94		60.32 ± 10.85	
Gender						
Male	156	69.96	173	67.58	329	68.68
Female	67	30.04	83	32.42	150	31.32
Smoking history						
Yes	152	68.16	159	62.11	311	64.93
No	71	31.84	97	37.89	168	35.07
Stage						
I-II	153	68.61	155	60.55	308	64.30
IIIA	70	31.39	101	39.45	171	35.70
Pathological tumor classification(pT)						
pT1-2	196	87.89	216	84.38	412	86.01
pT3-4	27	12.11	40	15.63	67	13.99
Pathological lymph node stage(pN)						
pN0	134	60.09	128	50.00	262	54.70
pN1-2	89	39.91	128	50.00	217	45.30
Histological type						
Squamous cell carcinoma	71	31.84	76	29.69	147	30.69
Adenocarcinoma	137	61.43	154	60.16	291	60.75
Large cell carcinoma	15	6.73	26	10.16	41	8.56
Surgery type						
Lobectomy	170	76.23	174	67.97	344	71.82
Pneumonectomy	41	18.39	62	24.22	103	21.50
Other	12	5.38	20	7.81	32	6.68
Adjuvant chemotherapy						
Yes	116	52.02	161	62.89	277	57.83
No	107	47.98	95	37.11	202	42.17
TG/HDL-C	1.16 ± 0.93		1.27 ± 0.87		1.22 ± 0.90	

Continuous variables with normality were presented as mean ± standard deviation. Categorical variables were shown as percentages.

**Table 2:** Correlations between TG/HDL-C and multiple clinical parameters in NSCLC patients.

Characteristics	TG/HDL-C								
	Training Cohort (*N* = 223)			Validation Cohort (*N* = 256)			Combined Cohort (*N* = 479)		
	Low	High	*p* Value	Low	High	*p* Value	Low	High	*p* Value
Age (years)			0.500			0.059			0.357
≤60	49	50		72	53		121	103	
>60	67	57		60	71		127	128	
Gender			0.404			0.455			0.264
Male	84	72		92	81		176	153	
Female	32	35		40	43		72	78	
Smoking history			0.985			0.437			0.568
Yes	79	73		85	74		164	147	
No	37	34		47	50		84	84	
Stage			0.064			0.431			0.069
I-II	86	67		83	72		169	139	
IIIA	30	40		49	52		79	92	
Pathological tumor classification(pT)			0.985			0.636			0.730
pT1-2	102	94		110	106		212	200	
pT3-4	14	13		22	18		36	31	
Pathological lymph node stage(pN)			0.002			0.006			<0.001
pN0	81	53		77	51		158	104	
pN1-2	35	54		55	73		90	127	
Histological type			0.038			0.609			0.331
Squamous cell carcinoma	45	26		36	40		81	66	
Adenocarcinoma	62	75		81	73		143	148	
Large cell carcinoma	9	6		15	11		24	17	
Surgery type			0.496			0.348			0.179
Lobectomy	92	78		94	80		186	158	
Pneumonectomy	18	23		27	35		45	58	
Other	6	6		11	9		17	15	
Adjuvant chemotherapy			0.152			0.194			0.054
Yes	55	61		78	83		133	144	
No	61	46		54	41		115	87	

Data were present with chi-square test; *p* < 0.05 was considered significant.

**Table 3:** Univariate and multivariate Cox proportional hazards model analyses for overall survival in the training and validation cohorts.

Characteristics	Training Cohort						Validation Cohort					
	Univariate Analysis			Multivariate Analysis			Univariate Analysis			Multivariate Analysis		
	HR	95%CI	*p* Value	HR	95%CI	*p* Value	HR	95%CI	*p* Value	HR	95%CI	*p* Value
Age (years)			0.127						0.412			
≤60	1.000	Reference					1.000	Reference				
>60	1.370	0.914–2.054					1.176	0.798–1.732				
Gender			0.281						0.802			
Male	1.000	Reference					1.000	Reference				
Female	0.781	0.499–1.224					0.949	0.628–1.434				
Smoking history			0.783						0.417			
Yes	1.000	Reference					1.000	Reference				
No	0.942	0.615–1.442					0.846	0.564–1.268				
Stage			<0.001			0.003			<0.001			0.029
I-II	1.000	Reference		1.000	Reference		1.000	Reference		1.000	Reference	
IIIA	4.289	2.854–6.444		2.734	1.401–5.334		3.553	2.380–5.303		2.196	1.086–4.439	
Pathological tumor classification(pT)			0.002			0.840			0.118			
pT1-2	1.000	Reference		1.000	Reference		1.000	Reference				
pT3-4	2.269	1.357–3.795		1.071	0.552–2.076		1.477	0.906–2.409				
Pathological lymph node stage(pN)			<0.001			0.001			<0.001			<0.001
pN0	1.000	Reference		1.000	Reference		1.000	Reference		1.000	Reference	
pN1-2	3.531	2.341–5.326		3.360	1.608–7.021		4.519	2.878–7.096		4.894	2.386–8.039	
Histological type			0.184						0.367			
Squamous cell carcinoma	1.000	Reference					1.000	Reference				
Adenocarcinoma	1.008	0.647–1.571					0.899	0.581–1.391				
Large cell carcinoma	1.920	0.906–4.068					1.404	0.718–2.743				
Surgery type			<0.001			0.086			<0.001			0.109
Lobectomy	1.000	Reference		1.000	Reference		1.000	Reference		1.000	Reference	
Pneumonectomy	3.364	2.146–5.274		1.967	1.048–3.693		2.605	1.728–3.927		1.379	0.797–2.386	
Other	3.329	1.643–6.743		1.288	0.508–3.264		1.525	0.727–3.199		0.633	0.275–1.455	
Adjuvant chemotherapy			0.042			<0.001			0.001			0.008
Yes	1.000	Reference		1.000	Reference		1.000	Reference		1.000	Reference	
No	1.519	1.014–2.275		3.616	1.872–6.987		2.155	1.386–3.350		2.803	1.306–6.017	
TG/HDL-C			0.001			0.017			<0.001			0.007
Low	1.000	Reference		1.000	Reference		1.000	Reference		1.000	Reference	
High	1.974	1.317–2.961		1.674	1.094–2.559		2.236	1.499–3.336		1.770	1.173–2.672	

Data were analyzed by Cox proportional hazards model; *p* < 0.05 was considered significant.

**Table 4:** Univariate and multivariate Cox proportional hazards model analyses for overall survival in the combined cohorts.

Characteristics	Combined Cohort					
	Univariate Analysis			Multivariate Analysis		
	HR	95%CI	*p* Value	HR	95%CI	*p* Value
Age (years)			0.097			
≤60	1.000	Reference				
>60	1.267	0.958–1.675				
Gender			0.337			
Male	1.000	Reference				
Female	0.862	0.636–1.167				
Smoking history			0.416			
Yes	1.000	Reference				
No	0.885	0.660–1.187				
Stage			<0.001			<0.001
I-II	1.000	Reference		1.000	Reference	
IIIA	3.789	2.851–5.035		2.480	1.518–4.050	
Pathological tumor classification(pT)			0.002			0.619
pT1-2	1.000	Reference		1.000	Reference	
pT3-4	1.756	1.233–2.502		1.117	0.723–1.724	
Pathological lymph node stage(pN)			<0.001			<0.001
pN0	1.000	Reference		1.000	Reference	
pN1-2	3.877	2.872–5.235		4.224	2.558–6.976	
Histological type			0.097			
Squamous cell carcinoma	1.000	Reference				
Adenocarcinoma	0.946	0.693–1.292				
Large cell carcinoma	1.578	0.958–2.598				
Surgery type			<0.001			0.046
Lobectomy	1.000	Reference		1.000	Reference	
Pneumonectomy	2.848	2.106–3.851		1.545	0.987–2.418	
Other	2.107	1.266–3.509		0.864	0.477–1.563	
Adjuvant chemotherapy			<0.001			<0.001
Yes	1.000	Reference		1.000	Reference	
No	1.767	1.317–2.370		3.486	2.113–5.754	
TG/HDL-C			<0.001			<0.001
Low	1.000	Reference		1.000	Reference	
High	2.108	1.586–2.801		1.715	1.279–2.301	

Data were analyzed by Cox proportional hazards model; *p* < 0.05 was considered significant.

## Data Availability

The data that supports the findings of this study are available on request from the corresponding author. The data are not publicly available due to privacy or ethical restrictions.

## Abbreviations

NSCLC: non-small cell lung cancer; TG: triglyceride; HDL-C: high-density lipoproteins cholesterol; TG/HDL-C: triglyceride to high-density lipoprotein cholesterol ratio; OS: overall survival; TNM: tumor-node-metastasis; UICC: Union for International Cancer Control; pT: pathological tumor classification; pN: pathological lymph node stage; ROC: receiver operating characteristic curve; AUC: area under the receiver operating characteristic curve; HR: hazard ratio; CI: confidence interval; TNF-α: tumor necrosis factor α; IL: interleukin.
